# Preliminary Results of Robotic Lobectomy in Stage IIIA-N2 NSCLC after Induction Treatment: A Case Control Study

**DOI:** 10.3390/jcm10163465

**Published:** 2021-08-05

**Authors:** Monica Casiraghi, Francesco Petrella, Giulia Sedda, Antonio Mazzella, Juliana Guarize, Patrick Maisonneuve, Filippo De Marinis, Lorenzo Spaggiari

**Affiliations:** 1 Division of Thoracic Surgery, IEO, European Institute of Oncology IRCCS, 20141 Milan, Italy; francesco.petrella@ieo.it (F.P.); giulia.sedda@ieo.it (G.S.); antonio.mazzella@ieo.it (A.M.); juliana.guarize@ieo.it (J.G.); lorenzo.spaggiari@ieo.it (L.S.); 2 Department of Oncology and Hemato-Oncology, University of Milan, 20122 Milan, Italy; 3 Division of Epidemiology and Biostatistics, IEO, European Institute of Oncology IRCCS, 20141 Milan, Italy; patrick.maisonneuve@ieo.it; 4 Division of Oncology, IEO, European Institute of Oncology IRCCS, 20141 Milan, Italy; fiippo.demarinis@ieo.it

**Keywords:** lung cancer, induction therapy, robotic surgery

## Abstract

Despite there already being many studies on robotic surgery as a minimally invasive approach for non-small-cell lung cancer (NSCLC) patients, the use of this technique for stage III disease is still poorly described. These are the preliminary results of our prospective study on the safety and effectiveness of robotic approaches in patients with locally advanced NSCLC in terms of postoperative complications and oncological outcomes. Since 2016, we prospectively investigated 19 consecutive patients with NSCLC stage IIIA-pN2 (diagnosed by EBUS-TBNA) who underwent lobectomy and radical lymph node dissection with robotic approaches after induction treatment. Furthermore, we matched a case-control study with 46 patients treated with open surgery during the same period of time, with similar age, comorbidities, clinical stage and tumor size. The individual matched population was composed of 16 robot-assisted thoracic surgeries and 16 patients who underwent open surgery. The median time range of resection was inferior in the open group compared to robotic lobectomy (243 vs. 161 min; *p* < 0.001). Lymph node resection and positivity were not significantly different (*p* = 0.96 and *p* = 0.57, respectively). Moreover, no difference was observed for PFS (*p* = 0.16) or OS (*p* = 0.41). In conclusion, we demonstrated that the early outcomes and oncological results of N2-patients after robotic lobectomy were similar to those who had open surgery. Considering the advantages of minimally invasive surgery, robot-assisted lobectomy appears to be a safe approach to patients with locally advanced diseases.

## 1. Introduction

Lung cancer represents the most frequent cause of death from malignancy worldwide, with non-small-cell lung cancer (NSCLC) as the most frequently presented type of epithelial lung cancer [1]. Approximately 10% of new diagnoses are patients with stage IIIA disease, with a mediastinal ipsilateral lymph node involvement (N2). In select cases, these patients may be candidates for surgery; however, considering the heterogeneity of patients, multimodality approach should always be considered. Indeed, the mediastinal involvement can range from limited N2 patients (with metastasis in only unilaterally mediastinal lymph nodes) to multi-station or bulky disease [2]. These variances can explain the different therapeutic approaches and oncological results, mirroring the complexity of locally advanced patients.

For potentially operable N2 disease, induction therapy (IT) followed by surgical resection has been demonstrated to improve overall survival (OS) and progression-free survival (PFS) [3]. Recently, we have shown that patients that are candidates for surgery after IT had a five-year OS that was significantly higher than those who underwent exploratory thoracotomy or incomplete resection (35% vs. 8% at five years, respectively). This underlines the importance of surgical resection of the residual disease in select patients [3].

In the last decades, several studies demonstrated the usefulness of the minimally invasive robotic-assisted thoracic surgery (RATS) in the treatment of lung cancer [4,5]; however, its use for N2 disease after IT is still debated, and the data are limited [6,7,8]. 

In this study, we present our preliminary results on the prospective evaluation of the safety and effectiveness of robot-assisted lobectomy in pN2-NSCLC patients after IT.

## 2. Materials and Methods

From January 1998 to May 2020, we performed 436 lung resections for pN2-IIIA after IT, of which 46 cases (10.5%) were performed by RATS (since 2006).

Since 2016, we prospectively analyzed all consecutive pathologically confirmed N2 NSCLC undergoing a RATS lobectomy (after IT) using a standardized questionnaire and protocol. The IEO Ethical Committee approved and deliberated the study in February 2017 (referred number IEO560). The primary point of the study was to include the evaluation of in-hospital morbidity (minor and major complication) and mortality, and peri- and post-operative outcomes, including operating time, amount of bleeding, duration of thoracic drainage, and days of hospitalization. The secondary endpoint was the OS and disease-free survival (DFS) and the percentage of patients with local and distant recurrence.

Nineteen out of forty-six (41.3%) patients undergoing a RATS lobectomy after IT met the inclusion criteria: diagnosis of NSCLC clinical stage IIIA for N2 with pathological confirmation of nodal involvement by endobronchial ultrasound-trans needle aspiration (EBUS-TBNA); IT with platinum-based chemotherapy (at least 2–3 cycles) or molecular therapy in patients with EGFR mutation (exon 19–20); staged by total-body computed tomography scan (CT) and fluorodeoxyglucose positron emission tomography (PET) with no evidence of distant metastasis. The exclusion criteria were: progression after induction therapy (bulky N2, or infiltration of surrounding mediastinal structures, multiple N2 stations, N3 disease or distant metastasis), patients unfit for surgery, previous thoracic surgery on the same side, and pneumonectomy or extended resection as a primary aim (hilar lesion or size of the tumor greater than 6 cm).

Next, we performed a match case-control analysis between 19 RATS patients and 46 patients undergoing open lobectomy for stage N2-IIIA NSCLC in the same period, with similar clinical and demographical characteristics.

### 2.1. Pre-Operative Evaluation

All patients underwent a preoperative evaluation, including CT, PET and cardiorespiratory assessment. All patients underwent mediastinal staging by EBUS-TBNA [9].

### 2.2. Surgical Procedure

All patients underwent anatomical lung resection and radical lymphadenectomy, according to the classification of the American Thoracic Society.

The RATS lobectomy was performed as previously published [5,10,11]. Briefly, the patient was positioned in a lateral decubitus, with the robot positioned at the head. The approach included three-port incisions and a 3 cm utility thoracotomy anteriorly in the IV intercostal space (however, the opening was performed in the IV and V), with no rib spreading. The camera was introduced in the VII intercostal space on the midaxillary line. The robotic instruments were inserted through the utility incision anteriorly and through the two posterior ports in the VIII and VII intercostal spaces, respectively. The robot was driven over the patient’s shoulder at a 15° angle and attached to the four ports. The ports were standard for all lobectomies except that, on the right side, the camera port through the VII intercostal space was in the mid-axillary line, whereas on the left side, this port was moved 2 cm posteriorly (compared with the right) to avoid the heart obscuring the vision of the hilar structures. 

### 2.3. Post-Intervention and Follow-Up Period

Patients were admitted to an intensive care unit (ICU) for one day after surgery, only if required by the anesthesiologist, based on the American Society of Anesthesiologists (ASA) score. Post-operative pain control during the hospital stay was managed with patient-controlled morphine administration, supplemented with intravenous analgesia. Oral analgesia was subsequently used when the patient was discharged.

Patients were followed with a physical exam, chest X-ray and blood tests at one month post-surgery, and with a physical exam plus CT scan of the chest and upper abdomen every four months for three years, then every six months until the fifth year, and annually after five years.

Recurrence at the site of surgery (hilar/mediastinal region or lung parenchyma closed to the previous resection), in the chest cavity (ipsilateral and contralateral such as new pulmonary nodules or pleural diffusion) and distant metastasis, were recorded and classified as local, regional and distant, respectively.

### 2.4. Statistical Analysis

The evaluation of primary endpoints (in-hospital morbidity and mortality, and peri and post-operative outcomes), including operating time, amount of bleeding, duration of thoracic drainage, and the days of hospitalization, were made using descriptive statistics (i.e., 95% confidence intervals for categorical variables, mean ± standard deviation, median and range for continuous variables). Survival analysis using the Kaplan-Meier curve and cumulative incidence analysis will evaluate the secondary endpoints (OS and DFS, development of local and distant relapses). In order to evaluate the outcome, we performed a matched case-control study: patients who had a RATS lobectomy were individually matched with patients treated with open surgery who had the same or similar clinical-stage, including tumor size, comorbidities and age. The McNemar test of symmetry and the non-parametric sign rank test were used to assess differences in the distribution of categorical and continuous characteristics between the two paired groups. The log-rank test and Gray’s test, respectively, were used to assess differences in OS and the DFS between the two groups. All analyses were 2-tailed; *p*-values were considered significant when *p* > 0.05.

## 3. Results

Clinical-demographic characteristics of the 19 patients enrolled in the study are shown in Table 1.

Based on the clinical staging with CT scan and PET, all patients underwent EBUS-TBNA mediastinal staging in case of suspected mediastinal lymph node involvement. The clinical-stage was pN2-pathologically proven-IIIA in all 19 (100%).

After multidisciplinary discussion, patients were subjected to IT: 17 (89.5%) underwent platinum-based chemotherapy, and 2 (10.5%) patients underwent only target therapy (one with Afatinib and one with Nivolumab, considering Epidermal growth factor receptor (EGFR) mutation).

All patients were re-staged within five weeks before the surgical procedure to confirm the feasibility of surgical resection. Two patients were converted to open surgery: one for oncological reasons, requiring extended resection, and one for adhesions.

The surgical and pathological features of all 19 patients are described in Table 2.

The majority of the patients underwent right upper lobectomies (36.8%), followed by right inferior lobe resection (26.3%). The median time of skin-to-skin RATS surgery was 266 min (153–420). The median size of the tumor was 20 mm (range 5–38 mm), and all patients had a complete resection (R0). The median number of lymph nodes harvested was 21 (range 6–52): 13 (range 2–40) N2, and 11 (0–28) N1. Furthermore, the median number of total lymph nodes stationed was 7 (range 4–9).

Minor post-operative complications occurred in four patients (21.1%): two with prolonged air-leaking, one with anemia and one with fever, treated with antibiotics. The median time of chest tube was four days (range 2–5) with a median length of hospital stay of six days (range 3–8).

Post-operative treatment was indicated in ten patients (52.6%): mediastinal radiotherapy was performed in nine patients (47.4%) for N2 persistent disease, whereas one patient underwent post-operative chemotherapy plus radiotherapy (5.3%). 

The median follow-up was 23 months (range 1–90 months). Recurrences were detected in six patients (31.6%): three lymph node relapses (N2 relapses) and three distant metastases (two brain and one liver metastasis).

### Matched Population

The individual matched population was composed of 16 RATS lobectomies and 16 patients undergoing open surgery; all matched patients were clinical stage IIIA-pN2 disease (confirmed by EBUS-TBNA) and comparable for age, comorbidities, clinical stage and tumor size (Table 1).

The median time of resection was inferior in the open group compared to the RATS lobectomies (161 vs. 243 min; *p* = 0.0007). The mean number of lymph nodes resected was 24 in both groups, and even the mean number of positive lymph nodes was not significantly different (*n* = 3.5 and 2.8, respectively; *p* = 0.57) between the two groups. The mean duration of ICU and hospital stay were comparable (*p* = 0.3 and *p* = 0.55, respectively), as well as the post-operative complication rate (*p* = 1.00) (Table 2). 

Considering the pathological stage, six (18.8%) patients in the RATS group had a confirmed single pN2 involvement compared to the two (6.3%) patients in the open group. Eight patients in the RATS group, and twelve in the open group, had persistence of ypN2 (50% and 75%, respectively); eight (50%) downstagings were evident in the RATS group (two cases to ypN1, and six to yN0) compare to four (25%) cases in the open one (one case to yN1 and three to yN0) (*p* = 0.42).

Post-operative mediastinal radiotherapy (RT) was administered in the case of persistent N2 disease in seven (43.8%) patients in the RATS group and eleven (68.8%) patients in the open group.

In the RATS group, a total of five patients (31.3%) developed recurrence of the disease: two had local recurrence with mediastinal involvement (12.5%), one regional (6.3%) and two distant metastases (12.5%). In the open group, twelve patients (75%) had post-operative relapses: one had local recurrence with mediastinal involvement (6.3%), three regional metastasis (18.8%) and eight distant metastasis (50.0%).

The median follow-up was 25 months (range 3–92). The majority of the patients were alive without any evidence of the disease: eight open patients (25.0%) and twelve in the RATS group (37.5%). Moreover, a total of six patients (three for each group, 9.4%) were alive with the disease, and one in the RATS (3.1%) and five in the open group (15.6%) died due to the disease’s progression.

**Figure 1:**
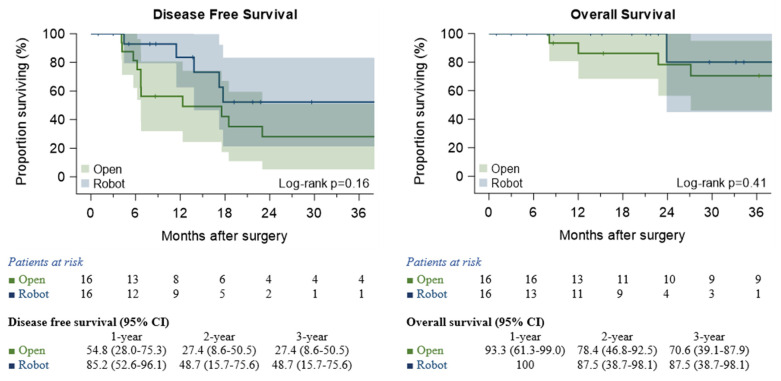
Disease-free survival and overall survival in the matched study. The median duration of observation was 23 months (range 1–90).

No difference was observed between the groups either in terms of DFS (Log-rank *p* = 0.16) or OS (Log-rank *p* = 0.41) (Figure 1).

## 4. Discussion

To date, VATS or RATS are the most common minimally invasive approaches in early-stage NSCLC (stage I and II) [5]. Despite some recent studies describing the use of robotic surgery in patients with both initial and locally advanced tumors [6,12,13,14,15], the results of this approach, specifically for stage III disease, have been demonstrated only in a very limited retrospective series [6,7,16,17,18,19], showing similar oncological outcomes between open and minimally invasive groups, both in terms of post-operative outcome and long-term survival. However, the use of the robotic approach after IT, even in these multicenter series’, is still limited to few cases [8,19].

Recently, Veronesi et al. published a retrospective multicenter study showing one of the largest series (*n* = 223) of pN2 lung cancer (NSCLC and carcinoid) patients undergoing RATS [8]. In this study, the author reported acceptable perioperative morbidity, with only 2.7% of cases being converted to open surgery due to emergency bleeding, showing the safety and feasibility of the procedure, even in the 34 patients who underwent IT. Thus, only four (12%) patients had grade III or IV post-operative complications. 

In fact, the lack of perception could be considered one of the major difficulties related to RATS, making the surgery particularly difficult, especially after IT or RT, with a higher risk of intra operative complications. However, in Veronesi’s study, only tumor size (large) and the number of positive lymph nodes (>2) were associated with a higher risk of conversion, whereas unexpected mediastinal nodal invasion and preoperative treatments were not related to any thoracotomy conversion. 

Moreover, in our study, even if our conversion rate was 10% (two cases out of nineteen), we did not have any cases due to bleeding, only one case for a bronchial infiltration of a pathological lymph node and another one for massive pleural adhesions. 

Veronesi [8] showed an estimated three-year survival in NSCLC patients of 61.2%, highlighting an excellent oncological outcome even if it could be related to the fact that most of the patients (*n* = 142/223; 63%) had occult N2 disease, and only 34 (15%) patients had IT. In a multicenter retrospective study, published in 2018, Cerfolio [19] analyzed 1339 patients undergoing RATS lobectomy, of which only 31 out of 122 pN2 NSCLC underwent IT (twenty-seven had induction chemotherapy, and four patients had induction CT/RT), showing an excellent intraoperative staging with a median number of lymph nodes harvested of 13 (five N2 stations and one N1). This allowed a greater discovery of occult N2 disease, which led to the use of adjuvant chemotherapy in 76% of these patients. Considering the long-term OS, Cerfolio showed an excellent 62% of five-year stage-specific survival for stage IIIA (73% in N2 disease). However, the authors highlighted how the survival dropped to 51% in patients undergoing IT, but it was better in patients who had adjuvant therapy (66%) after a correct surgical staging.

Even in our previous study of 339 RATS for early-stage NSCLC, we showed an excellent overall lymph node upstaging (17.6%), with 58% of five-year stage-specific survival for occult pN2 patients (*n* = 28) [5]. This confirmed that during RATS, mediastinal lymph nodes are adequately assessed, leading to excellent oncologic results. Furthermore, in this study, the mean number of lymph node resections was 24, in line with the literature findings and comparable to the open approach (*p* = 0.96) in the matched study.

Considering our median follow-up of 23 months, both DFS and OS were comparable in the two groups (Log-rank *p* = 0.16 and Log-rank *p* = 0.41, respectively). Unfortunately, our median follow-up is still too short, considering the preliminary nature of our results; thus, we cannot yet make any oncological or long-term considerations. However, among the worldwide guidelines [20] and the already established consensus on the surgical resection of N2-NSCLC after induction CT, robotic approaches could be considered as a valid alternative in the complex decision-making algorithm for N2-disease management, considering that RATS allows an adequate assessment of the lymph node, essential for oncological outcomes, as well as the proven advantages in terms of quality of life. Besides, in the era of personalized medicine and the introduction of immunotherapy, the management of N2-disease could rapidly change even more in favor of the surgical approach, due to a better pathological response, despite the major technical difficulties related to the inflammatory response. Thanks to a better vision and excellent instrument maneuverability of robotic instruments compared to VATS, a RATS lobectomy appears feasible in expert hands.

The major biases of this study are the small number of patients evaluated and the short follow-up, which limit and influence the long-term results. Besides, another important bias could be related to the case selection for the RATS group; in a real clinical situation, it is almost impossible to avoid selection bias for the early phase of a new approach, where the simple cases would be chosen preferentially, not only for the operative success rate but for the patient’s interest. 

## 5. Conclusions

Considering the advantages of minimally invasive surgery, our data suggest that RATS lobectomies should be a valid alternative after induction treatment, with comparably favorable prognoses to open surgery. However, these results require prospective studies (such as our ongoing multicenter prospective study for stage IIIApN2 NSCLC) and further testing on a larger population to be validated.

## Figures and Tables

**Table 1:** Clinical and demographical characteristics of all patients and matched patients.

	Unmatched			Matched		
	Robot	Open	*p*-Value	Robot	Open	*p*-Value
**Total**	19 (100.0)	46 (100.0)		16 (100.0)	16 (100.0)	
**Age**						
<60	7 (36.8)	13 (28.3)		5 (31.3)	8 (50.0)	
60–69	8 (42.1)	17 (37.0)		7 (43.8)	4 (25.0)	
70+	4 (21.1)	16 (34.8)	0.62	3 (18.8)	4 (25.0)	0.65
**Sex**						
Male	8 (42.1)	28 (60.9)		6 (37.5)	9 (56.3)	
Female	11 (57.9)	18 (39.1)	0.18	10 (62.5)	7 (43.8)	0.48
**Comorbidities**						
Cardiovascular disease	9 (47.4)	19 (41.3)	0.78	7 (43.8)	5 (31.3)	0.72
Pulmonary	1 (5.3)	3 (6.5)	1.00	1 (6.3)	0 (0.0)	1.00
Diabetes	1 (5.3)	5 (10.9)	0.66	1 (6.3)	2 (12.5)	1.00
**CT**						
1a	0 (0.0)	2 (4.3)		-	2 (12.5)	
1b	6 (31.6)	7 (15.2)		5 (31.3)	4 (25.0)	
1c	4 (21.1)	12 (26.1)	0.45	3 (18.8)	5 (31.3)	0.17
2a	2 (10.5)	11 (23.9)	Trend	2 (12.5)	4 (25.0)	Trend
2b	7 (36.8)	14 (30.4)	0.81	6 (37.5)	1 (6.3)	0.12
**N Station positive** **(EBUS-TBNA)**						
2R	0 (0.0)	0 (0.0)		0 (0.0)	0 (0.0)	
4R	10 (52.6)	30 (65.2)		8 (50)	10 (62.5)	
ST5	3 (15.8)	2 (4.3)		3 (18.8)	1 (6.3)	
ST6	0 (0.0)	1 (2.2)		0 (0.0)	0 (0.0)	
ST7	6 (31.6)	13 (28.3)	0.25	5 (31.2)	5 (31.2)	0.48
**Size, mm**						
mean ± SD	20 ± 10	30 ± 13	**0.004**	21 ± 10	22 ± 10	0.61

Bold text indicates a statistically significant difference with a *p*-value less than 0.05.

**Table 2:** Surgical and pathological characteristics of all patients and matched patients.

	Unmatched			Matched		
	Robot	Open	*p*-Value	Robot	Open	*p*-Value
**Total**	19 (100.0)	46 (100.0)		16 (100.0)	16 (100.0)	
**pT**						
ypT0	3 (15.8)	0 (0.0)		3 (18.8)	0 (0.0)	
ypT1	8 (42.1)	17 (37.0)	0.04	5 (31.3)	9 (56.3)	0.22
ypT2	7 (36.8)	21 (45.7)	Trend	7 (43.8)	5 (31.3)	Trend
ypT3	1 (5.3)	8 (17.4)	**0.02**	1 (6.3)	2 (12.5)	0.51
**pN**						
ypN0	7 (36.8)	6 (13.0)	0.08	6 (37.5)	3 (18.8)	
ypN1	2 (10.5)	5 (10.9)	Trend	2 (12.5)	1 (6.3)	
ypN2	10 (52.6)	35 (76.1)	**0.03**	8 (50.0)	12 (75.0)	0.42
**Grade**						
1	2 (10.5)	11 (23.9)	**0.02**	2 (12.5)	5 (31.3)	0.09
2	9 (47.4)	7 (15.2)	Trend	8 (50.0)	3 (18.8)	Trend
3	4 (21.1)	21 (45.7)	0.62	3 (18.8)	7 (43.8)	0.85
**FEV1 %**						
mean ± SD	95 ± 17	91 ± 19	0.43	98 ± 15	88 ± 18	0.08
**DLCO/VA**						
mean ± SD	82 ± 19	86 ± 20	0.42	83 ± 21	80 ± 16	0.75
**ASA score**						
2	13 (68.4)	35 (76.1)		11 (68.8)	12 (75.0)	
3	6 (31.6)	11 (23.9)	0.55	5 (31.3)	4 (25.0)	1.00
**Lymph node resected, *n***						
mean ± SD	23 ± 9	24 ± 11	0.81	24 ± 10	24 ± 10	0.96
**Lymph node positive, *n***						
mean ± SD	2.8 ± 3.8	3.6 ± 2.9	0.34	2.8 ± 4.0	3.5 ± 2.6	0.57
**Surgery, hours**						
mean ± SD	226 ± 55	165 ± 61	**0.0001**	243 ± 73	161 ± 42	**0.0007**
**ICU, days**						
mean ± SD	0.1 ± 0.2	0.1 ± 0.3	0.37	0.1 ± 0.3	0.2 ± 0.4	0.30
**Hospital stay, days**						
mean ± SD	5.2 ± 1.5	6.4 ± 4.34	0.08	5.1 ± 1.4	5.4 ± 1.5	0.55
**Complications**						
No	15 (78.9)	36 (78.3)		11 (68.8)	10 (62.5)	
Yes	4 (21.1)	10 (21.7)	1.00	3 (18.8)	4 (25.0)	1.00
Cardiovascular	1 (5.3)	6 (13.0)		0 (0.0)	3 (18.8)	
Pulmonary	2 (10.5)	5 (10.9)		2 (12.5)	1 (6.3)	
Surgical	0 (0.0)	3 (6.5)		0 (0.0)	2 (12.5)	
Other	1 (5.3)	0 (0.0)		1 (6.3)	0 (0.0)	
Minor	4 (21.1)	8 (17.4)		3 (18.8)	3 (18.8)	
Major	0 (0.0)	2 (4.3)		0 (0.0)	1 (6.3)	

FEV1%: forced expiratory volume in one second (% of predicted); SD: standard deviation; ICU: intensive care unit; DLCO/VA: diffusing capacity for carbon monoxide/alveolar volume; ASA: American Society of Anesthesiology. Bold text indicates a statistically significant difference with a *p*-value less than 0.05.

## Data Availability

The data presented in this study are available on request from the corresponding author.
